# Structural Dynamics and Perspectives of Vitamin B6 Biosynthesis Enzymes in *Plasmodium*: Advances and Open Questions

**DOI:** 10.3389/fcimb.2021.688380

**Published:** 2021-07-13

**Authors:** Angélica Luana C. Barra, Najeeb Ullah, Luana G. Morão, Carsten Wrenger, Christian Betzel, Alessandro S. Nascimento

**Affiliations:** ^1^ Pólo TerRa, São Carlos Institute of Physics, University of São Paulo, São Carlos, Brazil; ^2^ Institute of Biochemistry and Molecular Biology, Laboratory for Structural Biology of Infection and Inflammation, University of Hamburg, Hamburg, Germany; ^3^ Unit for Drug Discovery, Department of Parasitology, Institute of Biomedical Sciences, University of São Paulo, São Paulo, Brazil

**Keywords:** *Plasmodium falciparum*, malaria, pyridoxal 5-phosphate, vitamin B6, Pdx1, Pdx2

## Abstract

Malaria is still today one of the most concerning diseases, with 219 million infections in 2019, most of them in Sub-Saharan Africa and Latin America, causing approx. 409,000 deaths per year. Despite the tremendous advances in malaria treatment and prevention, there is still no vaccine for this disease yet available and the increasing parasite resistance to already existing drugs is becoming an alarming issue globally. In this context, several potential targets for the development of new drug candidates have been proposed and, among those, the *de novo* biosynthesis pathway for the B6 vitamin was identified to be a promising candidate. The reason behind its significance is the absence of the pathway in humans and its essential presence in the metabolism of major pathogenic organisms. The pathway consists of two enzymes i.e. Pdx1 (PLP synthase domain) and Pdx2 (glutaminase domain), the last constituting a transient and dynamic complex with Pdx1 as the prime player and harboring the catalytic center. In this review, we discuss the structural biology of Pdx1 and Pdx2, together with and the understanding of the PLP biosynthesis provided by the crystallographic data. We also highlight the existing evidence of the effect of PLP synthesis inhibition on parasite proliferation. The existing data provide a flourishing environment for the structure-based design and optimization of new substrate analogs that could serve as inhibitors or even suicide inhibitors.

## Introduction

According to the World Health Organization (WHO) Malaria Report from 2020 (World Health Organization, 2020), a total of 229 million malaria cases were reported in 2019 in 87 endemic countries, most of them in sub-Saharan Africa. Despite recent advances in the treatment, approx. 409,000 deaths still were reported in 2019 due to Malaria and, unfortunately, approx. 65% of these deaths are children less than 5 years old (World Health Organization, 2020). For comparison, the COVID-19 pandemic caused 450,000 deaths from April/2020 to the middle of January/2021 in South America and Africa together, suggesting that malaria is equivalent in terms of the number of deaths to COVID-19 pandemic for these regions. Although the malaria fatality rate is declining worldwide, it is obvious that substantial more efforts are required to more effectively treat malaria in the endemic areas.

The resistance of the major malarial pathogen *Plasmodium falciparum* to chloroquine and widely used sulfadoxine-pyrimethamine has been reported since the ‘50s and ‘60s of the last century. Even for the artemisinin-based combination therapy, recommended by the WHO to treat malaria (World Health Organization, 2015), an increasing resistance was observed in endemic areas, leading the WHO to launch the *Emergency Response to Artemisinin Resistance* (ERAR) in 2013. Adding to this, antimicrobial-resistant infections cause approx. 700,000 deaths annually worldwide, with an estimated increase of up to 10 million by the year 2050 (IACG, 2019). Concerning the severity of this situation, a forum of over 20 world’s leading pharmaceutical companies launched the establishment of the “AMR Action Fund” in 2020, with an investment goal to bring 2-4 new drugs into the market by the year 2030 (Clancy and Nguyen, 2020). Altogether, there has been an enormous effort from the scientific community for the identification of new molecular targets and, possibly, for the proposition of novel drug candidates that could be used in malaria-endemic areas.

**Figure 1:**
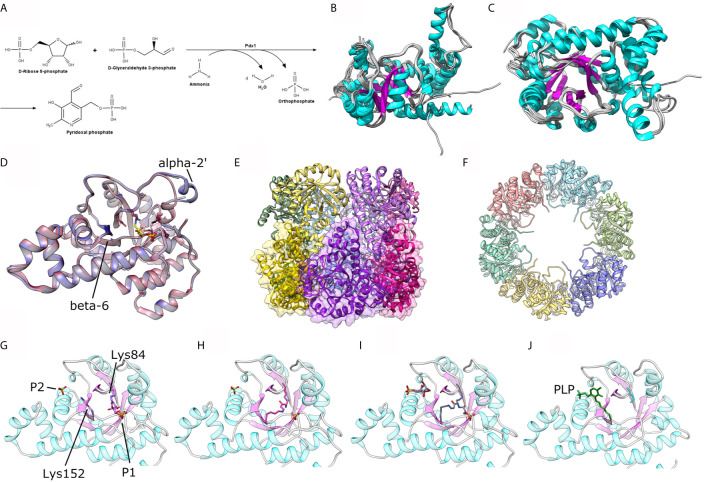
**(A)** Scheme for the enzymatic reaction catalyzed by Pdx1. **(B, C)** Two orthogonal views of a structural alignment of one representative structure of each organism, as available in the PDB for *P. berghei* (PDB ID: 4ADU), *A*. *thaliana* (5LNR), *T. maritima* (2ISS), *T. thermophilus* (2ZBT), *G*. *kaustophilus* (4WY0), *G*. *stearothermophilus* (1ZNN), *B. subtilis* (2NV1), *S. cerevisae* (3FEM), *M. tuberculosis* (4JDY), *M. jannaschii* (2YZR) and *P. horikoshii* (4FIQ). **(D)** Comparison between the crystal structure of *P. berghei* Pdx1 in the absence (red cartoon, PDB ID 4ADU) and presence (blue cartoon, PDB ID 4ADS) of Pdx2. **(E, F)** Two orthogonal views of the assembled Pdx1 dodecamer. The bottom hexamer is shown in **(E)** as cartoons and transparent surfaces. **(G, H)** Different steps in *A*. *thaliana* Pdx1 catalysis: **(G)** Pdx1 covalently attached to R5P; **(H)** Pdx1 intermediate I_320_; **(I)** Pdx1 intermediate I_320_-G3P; **(J)** Pdx1 covalently bound to the product PLP. The catalytic lysines are shown in sticks presentation and indicated in the figure. The figure was prepared with the program UCSF Chimera (Pettersen et al., 2004).

An interesting approach and potential drug target involve the *de novo* biosynthesis of vitamins in *Plasmodium*. In this context, Chan and coworkers showed that the inhibition of plasmodial thiamine pyrophosphokinase using the analog oxythiamine results in the inhibition of parasite proliferation (Chan et al., 2013). A similar approach was also evaluated for other essential pathways. Wrenger and coworkers identified the genes for the enzymes Pdx1, Pdx2, and PdxK in *Plasmodium falciparum*, responsible for the biosynthesis and salvage of pyridoxal 5’-phosphate (PLP) (Wrenger et al., 2005; Müller et al., 2010). In contrast to the biosynthesis pathway of thiamine, the *de novo* biosynthesis of PLP (vitamin B_6_) requires only two enzymes (Pdx1 and Pdx2) to form a complex (Pdx1-Pdx2 or PLP synthase complex) (Barra et al., 2020). The reaction catalyzed by the PLP synthase complex is shown in **Supplementary Figure S1**, while the individual reactions catalyzed by Pdx1 and Pdx2 are shown in **Figures 1A**, **3A**. In *Plasmodium*, the expression profile of the genes *pdx1* and *pdx2*, encoding Pdx1 and Pdx2, indicates that both proteins are localized in the cytosol (Wrenger et al., 2005).

*Plasmodium* depends on the *de novo* synthesis of the B6 vitamin as well as on the uptake of the non-phosphorylated vitamin from the environment. This conclusion is supported by the finding that *P. berghei pdx1* is necessary for optimal development of the parasite in the blood stage, although not essential for survival. Also, PdxK, the enzyme responsible for the B6 vitamin salvage, was not found to be essential (Derrer, 2010). However, a knock-out of the *pdx1*, *pdx2*, and *pdxk* genes was never obtained, suggesting that the *de novo* and salvage pathways are jointly necessary for parasite survival. In the context of human infection, the salvage pathway seems to be less necessary, since the available nutrients are mainly phosphorylated, and the *de novo* synthesis of the vitamin becomes more relevant.

Vitamin B6 has a dual role in *P. falciparum*. The classical role of PLP involves its electrophilic stabilizer effect of the carbanion in several enzymatic reactions. The PLP-dependent enzymes can be classified into seven groups, according to the enzyme activity: (*i*) aminotransferases and the amino-acid decarboxylases, such as serino hydroxymethyltransferases and aspartate aminotransferases; (*ii*) activity on the replacement and elimination of Cβ groups, such as serine and threonine dehydratases; (*iii*) activity on the interconversion of L- and D-amino acids with a common fold (α/β)_8_, such as alanine racemase; (*iv*) alanine aminotransferase; (*v*) glycogen phosphorylase; (*vi*) 5,6-aminomutase; and (*vii*) 2,3-aminomutase (Kronenberger et al., 2014). In *Plasmodium*, most of the PLP-dependent enzymes are classified in groups 1 and 2, highlighting their relevance in amino acid metabolism (Kappes et al., 2011; Kronenberger et al., 2014). A second role of PLP in *Plasmodium* involves the protection of the parasite against singlet oxygen (^1^O_2_). Knöckel and coworkers showed that the transcriptional levels of the genes *pdx1* and *pdx2* are up-regulated in the presence of cercosporin, as a response to oxidative stress (Knöckel et al., 2012). The authors also showed the protective effect of the overexpression of Pdx1 and Pdx2, resulting in an increased survival rate of the parasite in an oxidative environment (Knöckel et al., 2012).

A crucial aspect of the *de novo* PLP biosynthesis is whether the pathway can be considered as a target for drug discovery to treat malaria. Reeksting and coworkers reported that a hydrazide analog of D-erythrose 4-phosphate, 4PEHz, inhibited *P. falciparum* Pdx1-Pdx2 with an IC_50_ of 16 µM (Reeksting et al., 2013). The compound was also tested for its effect on parasite proliferation in the intra-erythrocytic cultures with an IC_50_ of 10.4 µM. It is also interesting to note that the 4PEHz effect was observed even in the culture medium containing 5 µM pyridoxine, suggesting that the inhibitory effect can overcome the salvage pathway (Reeksting et al., 2013). This proof-of-concept study strongly suggests that the enzymes involved in the PLP biosynthesis pathway should be further explored and considered as potential drug targets.

Ideal cases of novel drug development strategies include the inhibition of the pathogen’s drug targets specifically, with least or almost no toxicity to the human host (Lewis, 2012). Therefore, selected drug targets should be distinctly different from the host’s metabolic processes, or even absent in the host (Begum et al., 2013). In this context, the presence of the PLP synthesis enzymes in the metabolic pathways of the majority of organisms, including bacteria, fungi, protozoans, and plants, and their absence in humans (Barra et al., 2020) avoids the off-target binding and potential side effects.

In this focused review, we summarize the current status of ongoing research to characterize the PLP biosynthesis pathway in *Plasmodium* species, and the results obtained till now. Additionally, we briefly revise the medicinal chemistry perspectives for the pathway and summarize some remaining questions.

### PDX1 Structure-Function and Dynamics

The deoxyxylulose 5-phosphate (DXP)-independent (R5P-dependent) pathway, identified first in 1999, (Ehrenshaft et al., 1999), consists of two proteins Pdx1 and Pdx2 that dynamically assemble to form a transient 24-meric Pdx1-Pdx2 complex. The Pdx complex consists of a dodecameric Pdx1 core, assembled into two independent functional D6-symmetric hexamers stacked together (**Figure 1**) (Strohmeier et al., 2006). The interior of the dodecameric Pdx1 is lined with the active sites of 12 Pdx1 monomer subunits, and in the saturated complex, 12 Pdx2 subunits are studded at the exterior region of the Pdx1 dodecamer with their active sites located at the Pdx1-Pdx2 interface regions.

The Pdx1 enzyme fold resembles the classic (β/α)8 or TIM (Triosephosphate Isomerase) barrel fold, with 8 parallel β-strands constituting the inner core that alternates with 8 main α-helices and their insertions around the β-strands (Zhu et al., 2005). It catalyzes the conversion of D-ribose 5-phosphate (R5P), D-glyceraldehyde 3-phosphate (G3P), and ammonia into pyridoxal 5-phosphate, water, and phosphate (**Figure 1A**) all in a series of approx. 20 or more enzymatic steps. The mechanism over the enzyme reaction is quite intricate and includes ring-opening of the R5P, the formation of a covalent imine intermediary through a lysine residue in the active site, several eliminations and tautomerization reactions, and the formation of new covalent intermediates with one of the stable chromophoric intermediate (I_320_) (Hanes et al., 2008). Such a mechanism is rather uncommon in metabolic pathways and opens a new route for drug design investigations targeting particular Pdx1 of the PLP enzyme complex.

To date, a number of Pdx1 crystal structures have been reported for prokaryotes as well as eukaryotes, with oligomeric structures mainly in the dodecameric form and a few Pdx1 hexamers. Dodecameric Pdx1 structures are reported for *Plasmodium berghei*, PDB ID 4ADT, (Guédez et al., 2012), *Arabidopsis thaliana* (5LNS, 6HX3) (Robinson et al., 2016; Rodrigues et al., 2017; Robinson et al., 2019), *Thermotoga maritima* (2ISS) (Zein et al., 2006), *Geobacillus kaustophilus* (4WXZ) (Smith et al., 2015), *G. staerothermophilus* (1ZNN) (Zhu et al., 2005), *Bacillus subtilis* (2NV1) (Strohmeier et al., 2006) and *Mycobacterium tuberculosis* (4JDY) (Kim and Kim, 2013). On the other hand, the hexameric oligomer of Pdx1 is reported for *Saccharomyces cerevisiae* (3O06 and 3FEM) (Neuwirth et al., 2009; Zhang et al., 2010), and archaeon *Pyrococcus horikoshii* (4FIQ) (Matsuura et al., 2012). When compared by their ternary structures utilizing the online server DALI (Holm and Rosenström, 2010), the above-mentioned homologs result in Z-score values ranging from 47 to 36, with RMS deviation values of 0.5 to 1.3 Å, revealing remarkable structural conservation across different organisms, from bacteria to plants, including yeast and *Plasmodium*, and an average sequence identity of approx. 60% against the reference *Pb*Pdx1 structure. The structural conservation is shown by the structural alignment of 10 crystal structures in **Figures 1B, C**.

Concerning the oligomeric state of Pdx1, the plasmodial enzyme spontaneously forms dodecameric oligomers in solution (**Figures 1E, F**), as also confirmed by electron microscopy for *Pf*Pdx1 (Knöckel et al., 2009; Derrer et al., 2010; Guédez et al., 2012). Ullah et al. also reported the dodecameric solution state of *P. vivax* Pdx1, utilizing complementary biophysical, bioanalytical, and X-rays solution scattering (SAXS) techniques, highlighting that the dodecameric solution form is a functional prerequisite of plasmodial Pdx1 proteins (Ullah et al., 2020). The dodecameric form of plasmodial Pdx1 was also evidenced *via* the crystal structure from *Plasmodium berghei* (Guédez et al., 2012).

These results are in line with the observations that most bacterial Pdx1 proteins are found to be dodecameric (Strohmeier et al., 2006). The exception here is the Pdx1 from *Pyrococcus horikoshii*, found to be a hexamer in solution, as well as in the crystalline state. Further detailed structural investigations suggested that the insertion of 37 residues in the sequence of *P. horikoshii* Pdx1 (Matsuura et al., 2012) changed this equilibrium towards the hexameric form. The *Saccharomyces cerevisae* Pdx1 was also found in hexameric form, both in solution and crystalline state, and this observation was attributed to the insertion of a lysine residue (Lys177) spatially located at the hexamer interface region (Neuwirth et al., 2009).

The comparison between the native *P. berghei* Pdx1 crystal structure and its complex with a covalent intermediate and with Pdx2 reveals subtle structural differences that are relevant to Pdx1 function (Guédez et al., 2012). In the absence of a ligand/substrate and Pdx2, the short helix α2’ (located near to the Pdx1 catalytic center) is found to be disordered in the plasmodial Pdx1 structure. Upon entry and covalent binding of the substrate R5P to the active site residue lysine the helix is ordered and oriented in the suggested “closed-confirmation” (**Figure 1D**) (Guédez et al., 2012). This closed-conformation is proposed to protect the substrate intermediates at the active site of Pdx1 from solvent modification and allowing the enzyme to accept the neutral ammonia transported from Pdx2.

A similar structural rearrangement was also observed in the plasmodial Pdx1-Pdx2 complex, where the attachment of the Pdx2 enzyme, aligns the helix α2’ of Pdx1 in an “opened-conformation”. The position of the helix in this conformation is different from what was observed for the substrate binding and does not cover the Pdx1 phosphate binding (P1) site (Guédez et al., 2012). The open conformation allows the enzyme to accept the entry of ammonia in the active site of Pdx1 through the hydrophobic ammonia tunnel. These conformational changes lead to the assumption that binding of Pdx2 induces an adaptative effect on Pdx1 allowing substrate binding (Raschle et al., 2009).

A structural scheme of the catalytic mechanism and individual intermediates of Pdx1 PLP synthesis was reported *via* six crystal structures of *A. thaliana* Pdx1 enzyme at distinct steps of the catalysis (Rodrigues et al., 2017). The authors reported an imine intermediate between lysine 98 (Lys84 in PbPdx1) and the C1 of R5P (**Figure 1G**). The intermediate I_320_ is formed afterward, upon adding ammonium chloride, causing the displacement of a phosphate and the formation of a second imine with lysine166 (Lys152 in PbPdx1, **Figure 1H**). Also, the repositioning of Lys166 introduces a subtle conformational change in the β-strand β6, where this residue is located. Afterward, the addition of G3P resulted in an I_320_-G3P intermediate formation with the G3P phosphate group occupying the same phosphate-binding site as observed for R5P, at the P1 binding site (**Figure 1I**). The next step in the catalytic mechanism is the actual conversion of R5P and G3P into PLP, covalently bound to Pdx1 through Lys166. In this case, it creates a second binding site called P2 binding site, similarly to P1, with a phosphate ion occupying the site in absence of the product PLP (Rodrigues et al., 2017). The catalytic cycle involves changes in the conformation of the Lys98 and Lys166 side chains, as well as a repositioning of the β-strand β6 and the small α-helix α2’ (**Figure 1J**). The conformational changes observed in the cycle are better visualized in a video animation prepared by ‘*morphing*’ the six *A. thaliana* crystal structures. The video is available as **Supplementary Material**.

**Figure 2:**
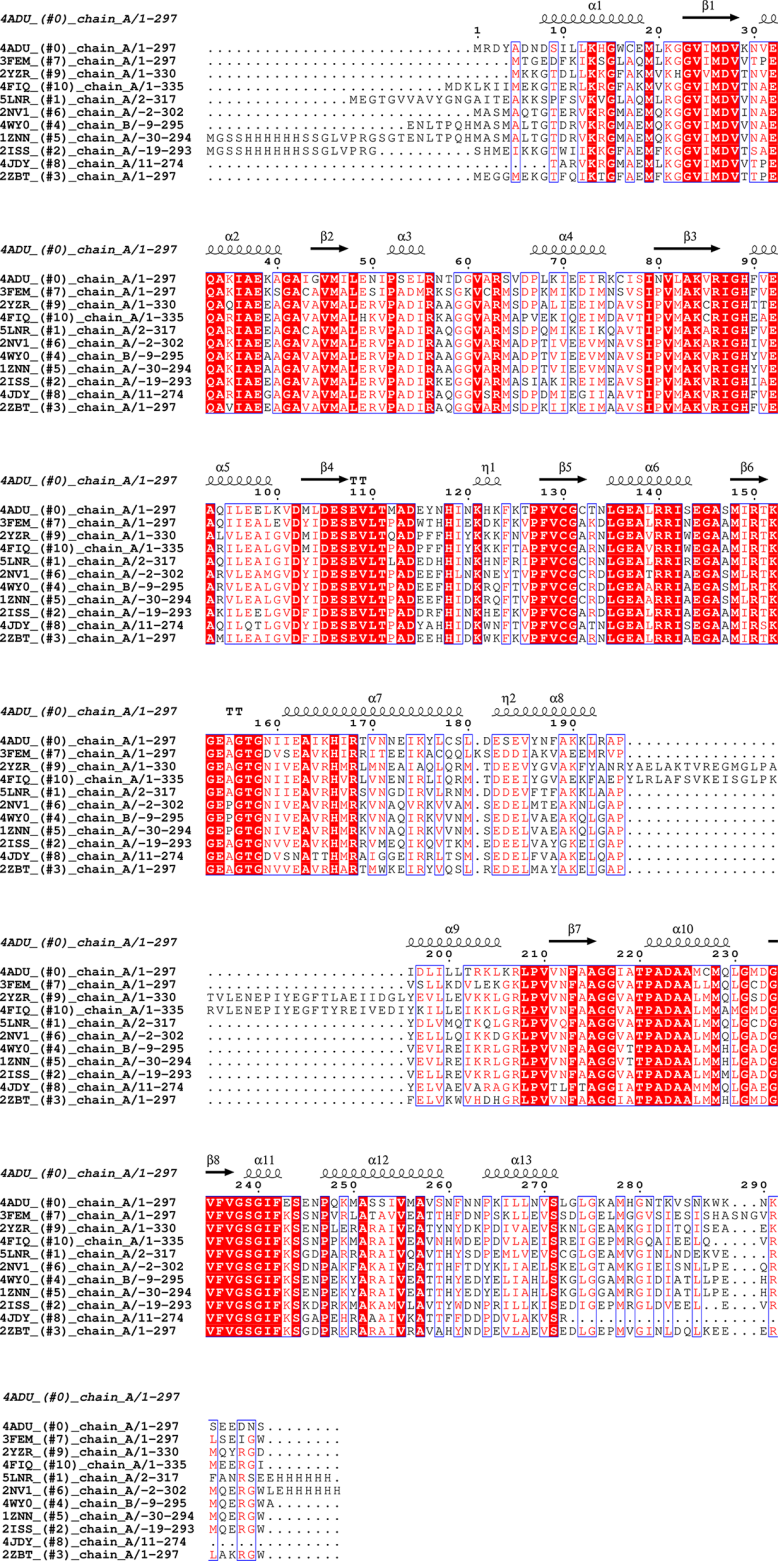
Sequence alignment for Pdx1 enzymes. Alignment prepared with UCSF Chimera (Pettersen et al., 2004) and EPPript (Robert and Gouet, 2014).

The P1 (involved in the accommodation of the substrates) and P2 binding sites (involved in product PLP formation and accommodation) are observed and reported in the Pdx1 monomer for several prokaryotes and eukaryotes: these sites are occupied by buffer salt anions in the absence of a substrate or product (Zhu et al., 2005). In terms of locations in the monomer barrel, the P1 site with characteristic GTG loop (^156^GTG in PbPdx1, **Figure 2**) is located in close spatial arrangement to the C-terminal side (Zhu et al., 2005). This site is usually occupied by chloride ions (Guédez et al., 2012) linked to other anions and mimicking the ribose substrate. The P2 site, distant approx. 15-20 Å from the P1 site, is usually occupied by chloride or phosphate ions in absence of enzyme ligands (Strohmeier et al., 2006; Rodrigues et al., 2017).

The analysis of the Pdx1 dodecamer from *P. berghei*, reveals that several of the residues involved in Pdx1 protomer interaction are located in conserved motifs across different species. These motifs include ^61^VxR, ^86^RIGHFVE, ^111^TxADx, ^140^RRISE, ^156^GTG, and ^181^DESE (PbPdx1 numbering), as shown in **Figure 2**. Interestingly, and in line with this observation, Knöckel and coworkers showed that the mutation G155A in *Pf*Pdx1 (Gly156 in *Pb*Pdx1, from the ^156^GTG motif) reduced the dodecamer equilibrium concentration and resulted in a hexamer formation in solution (Knöckel et al., 2009). Curiously, this mutant was incapable to synthesize PLP but was able to recruit and activate Pdx2 (Knöckel et al., 2009). Lys151 in *Pf*Pdx1 (K152 in *Pb*Pdx1), part of the active site and with an active role in catalysis, also showed similar behavior: the PfPdx1 K151A mutant was mainly hexameric and inactive, but able to recruit and activate *Pf*Pdx2 (Müller et al., 2008). Finally, the C-terminus of the Pdx1 is also directly involved in the oligomerization, as part of the interaction surface, although the sequence of this region is not conserved. To date, the reported data indicate that the C-terminus forms an extended loop, with spatial position adjacent to the loop α2´-α2 of the interface monomer, establishing inter-chain contacts for the Pdx1 hexamer formation (Neuwirth et al., 2009). This observation is also in line with the previous observation of Derrer and coworkers, who showed the involvement of the C-terminal and preceding helix α8’’ in Pdx1 dodecamer (Derrer et al., 2010).

The high-order oligomeric species observed in the PLP complex could suggest potential cooperativity in the synthesis of PLP. However, this does not seem to be the case when Pdx1 is analyzed in the absence of Pdx2. Typical hyperbolic curves are observed that seem to fit well to the classical Michaelis-Menten kinetic model (Wrenger et al., 2005), and no sigmoidal curve is observed in enzyme kinetics for most Pdx1 enzymes, as would be expected for a Hill cooperativity. Furthermore, it seems that *P. falciparum* PLP synthase activity is not affected by the presence or absence of Pdx2. The specific activity of PfPdx1 was determined as 746 pmol min^-1^ mg^-1^ when using ammonium chloride as the source of ammonia and 662 pmol min^-1^ mg^-1^ in the presence of Pdx2 and L-glutamine (Müller et al., 2008). Curiously, it is in contrast to what was observed for the *Mycobacterium tuberculosis* Pdx1 enzyme, where the substitution of Pdx2 by ammonium chloride resulted in a threefold reduction of the specific activity (Dick et al., 2010).

## Pdx2 Structure-Function and Dynamics

**Figure 3:**
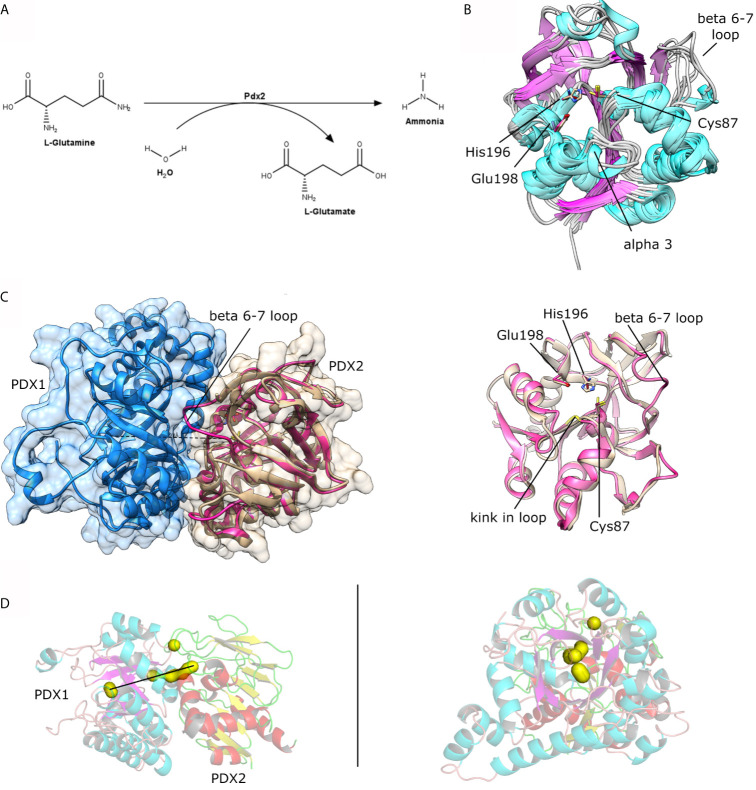
**(A)** Scheme for the enzymatic reaction catalyzed by Pdx2. **(B)** Structural superposition of the crystal structures as available in the PDB for *P. falciparum* Pdx2 (PDB ID 2ABW), *T. maritima* (2ISS), *G*. *kaustophilus* (4WXY), *B. stearothermophilus* (1Q7R), *B. subtilis* (2NV0), *M. jannaschii* (2YWJ). **(C)** Left, structural view of the Pdx1 (shown in blue cartoon and surface) and Pdx2 (shown in brown and pink cartoon presentation). The loop linking β-strands β6-β7 is indicated in the figure and is part of the interacting interface. Right, superposition of PfPdx2 in the absence (brown) and presence (pink) of Pdx1. **(D)** Two orthogonal views of the Pdx1-Pdx2 interaction. Some clefts in the complex are shown in yellow surface representation, indicating a putative way for the ammonia tunnel. The figure was prepared with UCSF Chimera (Pettersen et al., 2004) and the program PyMol.

Pdx2 is a glutaminase enzyme responsible for the conversion of glutamine into glutamate and ammonia, the last later delivered to Pdx1 for PLP synthesis (**Figure 3A** and **Supplementary Figure S1**). In *P. falciparum*, the *pdx1* and *pdx2* genes are located in chromosomes 11 and 6, respectively (Wrenger et al., 2005). It was observed that Pdx2 was inactive when expressed alone but exhibited glutaminase activity when mixed with Pdx1 in a 1:1 ratio (Wrenger et al., 2005; Gengenbacher et al., 2006). The enzyme specific activity was determined as 209 nmol min^-1^ mg^-1^, with a Michaelis-Menten constant of K_M_=1.3 mM for glutamine, according to Wrenger and coworkers (Wrenger et al., 2005), or a K_M_=0.56 mM and a k_cat_ of 0.11 s^-1^, according to Gengenbacher and coworkers (Gengenbacher et al., 2006). The PfPLP synthase complex accepts the five-carbon sugar R5P or ribulose-5-phosphate and three-carbon sugar G3P or dihydroxyacetone phosphate (DHAP) as substrates, with comparable activity. However, providing ammonium chloride in the buffer instead of Pdx2 as the ammonia source makes Pdx1 selective for G3P as a triose sugar (Gengenbacher et al., 2006).

The reasons for the Pdx1-based activation of Pdx2 were at least partially explained in the recent report from Ullah and coworkers (Ullah et al., 2020). The authors showed *via* dynamic light scattering (DLS) and X-ray solution scattering (SAXS) investigations that *P. vivax* Pdx2 has intrinsic flexible characteristics to form different oligomers over time. Further, they observed by time-resolved DLS and electron microscopy (EM) studies an interesting reversible oligomerization behavior of Pdx2, after mixing the multimeric/oligomeric Pdx2 with monodisperse dodecameric Pdx1. This indicates a stabilizing effect of the Pdx1 enzyme upon binding to Pdx2 and in this context for the Pdx complex (Ullah et al., 2020).

Pdx2 assembles with Pdx1 to form the PLP synthase complex spontaneously even in the absence of the enzyme substrates. Gengenbacher and coworkers described that the co-expression of both *P. falciparum* enzymes results in the spontaneous formation of the Pdx complex (Gengenbacher et al., 2006). The plasmodial PLP synthase complex formation was also observed by EM and SAXS studies (Guédez et al., 2012; Ullah et al., 2020). However, the saturation of Pdx1 enzymes with Pdx2 is only ensured in the presence of the substrate (Guédez et al., 2012), revealing that the activation by the substrate enhances the affinity and supports the interaction between Pdx1 and Pdx2. Additionally, they also observed that the active site mutation in the Pdx2 subunit (H196N), impaired the ability to metabolize glutamine, resulting in a saturated and fully decorated Pdx1-Pdx2 complex (Guédez et al., 2012). In another structural investigation of Pdx proteins from *Saccharomyces cerevisiae* the Pdx2 subunit was complexed without active site mutations in presence of the glutamine analog acivicin. The analog acivicin was detected in a covalently bound state to the catalytic cysteine residue (Chaudhuri et al., 2001), indicating inhibition of the Pdx2. These observations provide structural details to support the design of inhibitors in the context of drug discovery investigations to approach the PLP synthase complex with a focus on the native state of Pdx2.

The crystal structures of a few Pdx2 enzymes either alone or complexed with Pdx1, are reported at different evolutionary levels i.e. from *P. falciparum* (PDB code: 2ABW) (Gengenbacher et al., 2006; Guédez et al., 2012), *Thermotoga maritima* (PDB code: 2ISS) (Zein et al., 2006), *Geobacillus kautophilus* (PDB code: 4WXY) (Smith et al., 2015), *Bacillus subtilis* (PDB code: 1R9G, 2NV0) (Bauer et al., 2004; Strohmeier et al., 2006), and *M. jannaschii*, with significant differences between plasmodial and bacterial Pdx2 structures in their functional structural elements. The Pdx2 structure is classified as a Rossmann-Fold, with β-strands forming an extended central β-sheet core and α-helices surrounding the sheet architecture, producing a three-layered sandwiched structure, typical for the class I glutamine amidotransferase domain (Strohmeier et al., 2006).

**Figure 4:**
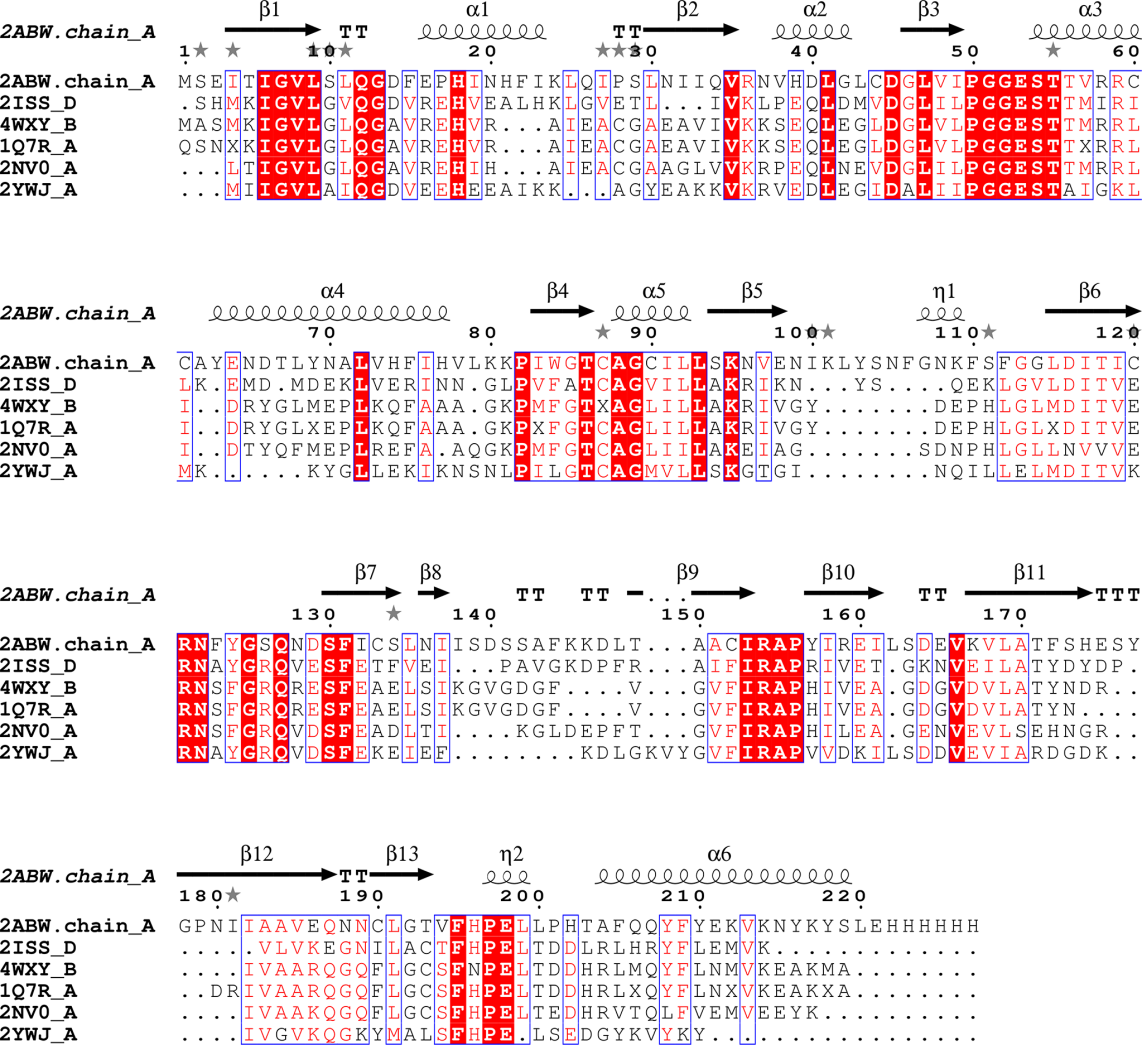
Sequence alignment for Pdx2 enzymes. Alignment prepared with UCSF Chimera (Pettersen et al., 2004) and EPPript (Robert and Gouet, 2014).

A search with the DALI server reveals that RMSDs compared between *Pf*Pdx2 and the enzymes mentioned before are in the range of 1.1 and 2.1 Å with Z-scores varying between 28 and 24. Interestingly, the typical sequence identity between the enzymes is approx. 38%, indicating structural conservation despite the divergence in sequence identity (**Figures 3B** and **4**).

The comparison between native *Pf*Pdx2 and the crystal structure of the enzyme in complex with *Pb*Pdx1 reveals structural adaptations of Pdx2 upon binding to its partner. The major rearrangement is the stabilization of the loop involving residues 123-128 (*Pf*Pdx2 numbering), connecting β6 and β7. This loop is missing in the higher-resolution structure of PfPdx2 and is stabilized in the PLP synthase complex, as shown in **Figure 3C**. This loop stabilization is usually observed in other glutamine amidotransferases (Mouilleron and Golinelli-Pimpaneau, 2007). Not surprisingly, the loop is located at the interface region between Pdx1 and Pdx2, interacting with the Pdx1 N-terminus helix. Another relevant function of the loop rearrangement is the formation of a solvent-excluded tunnel, connecting the active site of Pdx2 to the hydrophobic β-barrel of Pdx1, utilized by the nascent generated neutral ammonia molecule which diffuses to the active site of Pdx1. This ammonia tunnel is composed of hydrophobic residues (Guédez et al., 2012), connecting the synthase and glutaminase active sites of the Pdx complex, with both the active site distanced by approx. 25 Å (**Figure 3D**).

A second structural rearrangement observed in Pdx2 upon binding to Pdx1 is observed in the loop connecting β3-α3, within the conserved motif ^50^PGGEST and N-terminus region of helix α1 (**Figure 4**). The N-terminus part of the helix α1 is distorted, with a non-conserved Asp14 residue repositioned to a significant extent for more close spatial contact to the catalytic triad active site residues. The distance between the Cδ atoms in Asp14 and Glu198 changes from 9 Å in the native Pdx2 structure to 4 Å in the 3D structure of the Pdx1-Pdx2 complex, suggesting that Asp14 may act as an accessory residue during the enzyme catalysis. Additionally, a kink is introduced in the loop in the ^50^PGGEST motif, as shown in **Figure 3C**. This kink is introduced by a repositioning of Gly51, which moves about 2 Å when the position of the main chain nitrogen atom is compared in the bound and unbound states. This residue, together with Ala88 forms an oxyanion hole through the main chain amide nitrogen atoms. The oxyanion hole loop (prerequisite of glutaminase activity) is involved in the stabilization of the transient negative charge during the hydrolysis of glutamine (Gengenbacher et al., 2006; Mouilleron and Golinelli-Pimpaneau, 2007).

## From Structure-Function to Drug Design

As we mentioned before, examples of specific lead compounds designed to target the PLP synthase complex are still missing, to the best of the authors’ knowledge. So far, it is known that substrate analogs can inhibit the enzyme and, consequently, the parasite growth (Chaudhuri et al., 2001; Reeksting et al., 2013), although no further compound optimization has been done after this proof-of-concept. In this context, one could ask: ‘is the PLP synthase a viable druggable target’?

**Figure 5:**
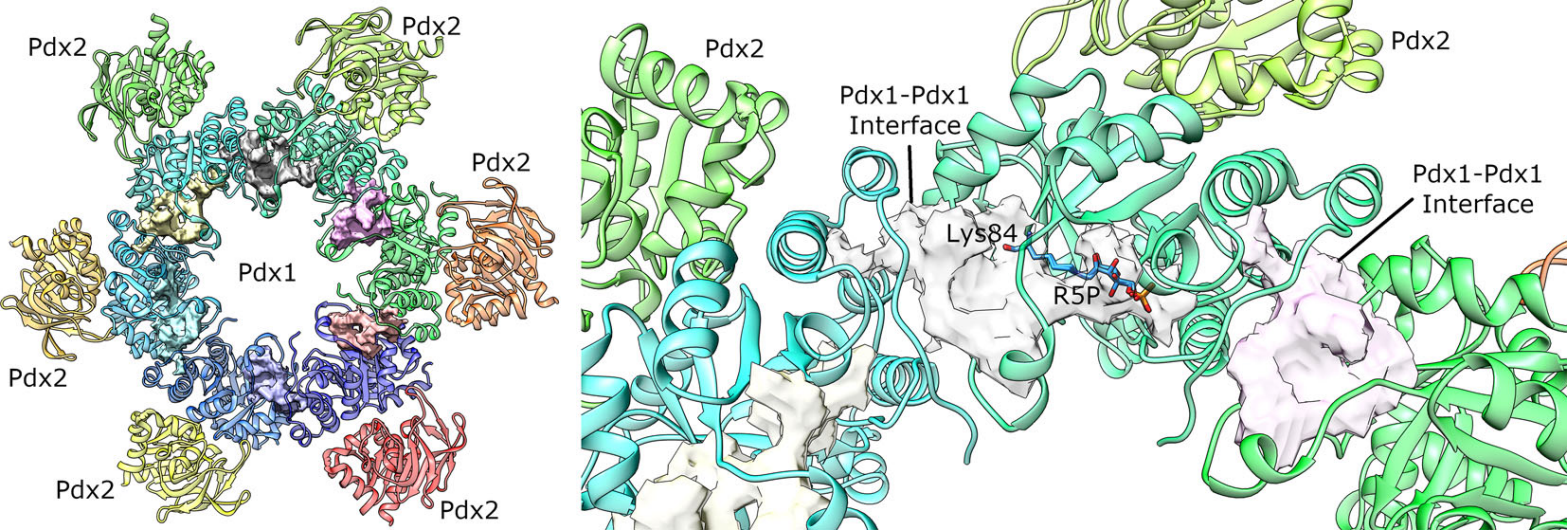
Pockets mapped on Plasmodial PLP synthase complex by DoGSiteScorer (Volkamer et al., 2012b). The larger identified pockets are shown as surfaces. (left) Overall view of the PLP synthase complex. The second Pdx1 hexamer and attached Pdx2 enzymes was omitted for clarity. (right) Detailed view of the best ranked pocket. For the sake of comparison, the substrate bound to the catalytic lysine 84 is also shown (manually docked from the *A. thaliana* crystal structure, PDB ID 5LNS). The surface in this site includes the substrate binding pocket and is also extended to the surface where the enzyme interacts with the partner shown in cyan ribbon. Similar pockets are also identified for the other monomers, as shown in pink and yellow transparent surfaces.

The DoGSiteScorer tool (Volkamer et al., 2012b; Volkamer et al., 2012a) matches a binding pocket that encompasses the active site (substrate binding site) but is also extended to include the interaction interface with the next monomer in the hexamer assembly (**Figure 5**). This pocket occupies a volume of approx. 1,800 Å^3^ and a DrugScore index of 0.81, suggesting the most suitable druggability of this site. The DrugStore is an index ranging from 0-1 and is estimated from a database of 1,069 structures, where DoGSiteScorer indicated an accuracy of 88% (Volkamer et al., 2012b). Interestingly, the analysis of the sites with the highest DrugScore indexes using SIENA (Bietz and Rarey, 2016) did not reveal any known similar pocket with an already known ligand comparable to the structural features of the PLP synthase complex.

Some of the pieces necessary for further advances in the proposal of the pyridoxine biosynthesis pathway as a druggable target are summarized in this focused review. **Supplementary Tables 1, 2** list the Pdx1 and Pdx2 crystal structures available by the time of this writing in the Protein Data Bank. We note that there are two plasmodial Pdx1 crystal structures with resolutions better than 2.5 Å and at least three crystal structures of non-plasmodial Pdx1 crystal structures with resolutions better than 1.7 Å (**Supplementary Table 1**). For Pdx2, a crystal structure of the plasmodial enzyme is available in the PDB with a 1.62 Å resolution, providing precise atomic details of the enzyme structure. This existing experimental data, provide the required basis for structure-based design and optimization of ligand candidates. Another piece comes from the *in vivo* activity of the substrate analogs on *Plasmodium* proliferation. This evidence is further complemented with the analysis of the druggability of the enzyme binding site. Putting the pieces together, one can easily envision a scenario where structure-based modeling is applied for the optimization of the substrate analogs and for the proposition of new compounds that could serve as chemical probes in the evaluation of the B6 vitamin pathway as an actual drug target.

PLP substrate analogs can be very interesting compounds in this context. Since the PLP synthase mechanism involves several covalent intermediates, these compounds could serve as *suicide inhibitors*, as previously exploited in other essential pathways (Drebes et al., 2014). An additional benefit of this approach is that it might be useful to other human pathogens since the enzyme active site is highly conserved.

The need for optimization of the existing inhibitor compound becomes obvious by an analysis of the similarity between 4PEHz and the known ligands of CHEMBL targets, as generated by the Similarity Ensemble Approach (SEA) (Keiser et al., 2007). The analysis shows that at least five human targets have a high probability of interaction with 4PEHz (*p*-value less than 1E^-27^), including 6-phosphogluconate dehydrogenase, Lysophosphatidic acid receptor 6, Triosephosphate isomerase, and Lysophosphatidic acid receptor 4 and 3. So, the structure-based optimization for this analog may be necessary to avoid off-targets.

## Discussion

Approx. 4% of all enzyme reactions listed by the Enzymatic Commission (EC) are PLP-dependent, highlighting the relevance of vitamin B6 to overall homeostasis (Percudani and Peracchi, 2009). The understanding of the plasmodial PLP *de novo* biosynthesis has advanced enormously in the past 15 years and many mechanistic aspects of the enzyme oligomerization and reaction are currently known. However, what is still missing? Certainly, a more complete validation of Pdx1 and Pdx2 as molecular targets in terms of drug discovery is still required, and more chemical probes (Gregori-Puigjané et al., 2012) are highly desirable. As we outlined, the PLP synthase, and particularly the Pdx1 enzyme, can be highly ranked as a druggable target according to chemoinformatic tools, but further efforts and investigations are still required.

The remaining open questions include: (*i*) is the interaction site between Pdx1 and Pdx2 druggable? (*ii*) Can PLP synthase activity be disrupted by Pdx2-specific inhibition? A piece of evidence in this context is the acivicin covalent inhibition of Pdx2, which resulted in the inhibition of parasite growth (Gengenbacher et al., 2006). However, acivicin is a known covalent inhibitor of glutaminases in general and the issue of a *specific inhibition* still awaits more and final investigation. (*iii*) Suicide inhibitors have been proposed in the context of vitamin *de novo* biosynthesis (Drebes et al., 2014), however, this approach is still now not yet evaluated for the PLP synthase pathway. To overcome the abovementioned challenges and corresponding questions will require the synergistic cooperation of different disciplines, such as biochemistry, structural and computational biology, parasitology, and medicinal chemistry.

## Author Contributions

All authors listed have made a substantial, direct, and intellectual contribution to the work, and approved it for publication.

## Funding

The authors acknowledge the financial support provided by Fundação de Amparo à Pesquisa do Estado de São Paulo (FAPESP) through grants 2015/26722-8, 2015/13684-0, 2020/03983-9, 2019/26428-3, 2019/20219-3, 2018/21213-6, 2017/03966-4 and by Conselho Nacional de Desenvolvimento Científico e Tecnológico (CNPQ) through grant number 303165/2018-9 and by the Cluster of Excellence ‘Advanced Imaging of Matter” Deutsche Forschungsgemeinschaft (DFG) - EXC 2056 - project 390715994, by BMBF *via* Project grants 05K19GU4, 05K20GUB and 05K16GUA. The authors also acknowledge the support grant from HEC, Pakistan, under the project “Faculty Development Program BZU Multan (100 Ph.D. Scholarships) (Prime Minister’s Directive)”.

## Conflict of Interest

The authors declare that the research was conducted in the absence of any commercial or financial relationships that could be construed as a potential conflict of interest.
