# Supplementary material for: Structural Dynamics and Perspectives of Vitamin B6 Biosynthesis Enzymes in Plasmodium: Advances and Open Questions
Source: Front Cell Infect Microbiol. 2021 Jul 13;11:688380. doi: 10.3389/fcimb.2021.688380 (PMC8313854; doi:10.3389/fcimb.2021.688380)
Supplement: Supplementary file 1 [file DataSheet_1.docx]

Structural Dynamics and Perspectives of Vitamin B6 Biosynthesis Enzymes in *Plasmodium*: Advances and Open Questions

Angélica Luana C. Barra^1,2¶^, Najeeb Ullah^2¶^, Luana G. Morão^1^, Carsten Wrenger^3*^, Christian Betzel^2*^, and Alessandro S. Nascimento^1*^.

1. Pólo TerRa, São Carlos Institute of Physics, University of São Paulo. Av. João Dagnone, 1100, Jd. Santa Angelina, 13563-120. São Carlos, SP. Brazil.

2. ﻿Institute of Biochemistry and Molecular Biology, Laboratory for Structural Biology of Infection and Inflammation, University of Hamburg, c/o DESY, Build. 22a. Notkestr. 85, 22603 Hamburg, Germany.

3. ﻿Unit for Drug Discovery, Department of Parasitology, Institute of Biomedical Sciences, University of São Paulo, Av. Prof. Lineu Prestes 1374, 05508-000 São Paulo-SP, Brazil.

* Corresponding authors: Phone: +55-16-3373-8709. Email: [asnascimento@ifsc.usp.br](mailto:asnascimento@ifsc.usp.br) (ASN) or Phone: +49 40 42838-6069. Email: [christian.betzel@uni-hamburg.de](mailto:christian.betzel@uni-hamburg.de) (C.B.) or Phone +55-11-2648-​8127. Email: [cwrenger@icb.usp.br](mailto:cwrenger@icb.usp.br).

^¶^ These authors contributed equally to this work.

| **Supplementary Table S1.** Pdx1 proteins with structure deposited on Protein Data Bank (PDB). | | | |
| --- | --- | --- | --- |
| Organism | PDB IDs | Resolution (Å) | Reference |
| *Plasmodium berghei* | 4ADT  4ADU  4ADS^ꝉ^ | 2.42  2.44  3.61 | Guédez et al., 2012 |
| *Arabidopsis thaliana* | 5LNR  5LNS  5LNU  5LNV  5LNW  5LNT  5K2Z  5K3V  6HYE  6HXG  6HX3 | 1.61  1.91  1.73  2.24  1.90  2.32  1.80  1.90  2.53  1.90  2.00 | Rodrigues et al., 2017  Robinson et al., 2016  Robinson et al., 2019 |
| *Thermotoga maritima* | 2ISS* | 2.90 | Zein et al., 2006 |
| *Thermus Thermophilus* | 2ZBT | 1.65 |  |
| *Geobacillus kaustophilus* | 4WY0  4WXZ  4WXY* | 2.30  2.70  2.70 | Smith et al., 2015 |
| *G. staerothermophilus* | 1ZNN | 2.2 | Zhu et al., 2005 |
| *Bacillus subtilis* | 2NV1  2NV2* | 2.08  2.12 | Strohmeier et al., 2006 |
| *Saccharomyces cerevisae* | 3FEM  3O05  3O06  3O07 | 3.02  2.20  2.35  1.80 | Neuwirth et al., 2009  Zhang et al., 2010 |
| *Mycobacterium tuberculosis* | 4JDY | 1.80 | Kim and Kim, 2013 |
| *Methanocaldococcus jannaschii* | 2YZR | 2.30 |  |
| *Pyrococcus horikoshii* | 4FIQ  4FIR | 2.70  3.10 | Matsuura et al., 2012 |
| * Pdx1-Pdx2 complex | | | |
| ^ꝉ^ PbPdx1 in complex with PfPdx2 | | | |

| **Supplementary Table S2.** Pdx2 proteins with structure deposited on Protein Data Bank (PDB). | | | | |
| --- | --- | --- | --- | --- |
| Organism | PDB IDs | | Resolution (Å) | Reference |
| *Plasmodium falciparum* | 2ABW  4ADS^ꝉ^ | | 1.62  3.61 | Gengenbacher et al., 2006  Guédez et al., 2012 |
| *Thermotoga maritima* | 2ISS* | | 2.90 | Zein et al., 2006 |
| *Geobacillus kaustophilus* | 4WXY* | | 2.70 | Smith et al., 2015 |
| *G. staerothermophilus* | 1Q7R | | 1.90 |  |
| *Bacillus subtilis* | 1R9G  2NV0 2NV2* | | 2.50  1.73  2.12 | Bauer et al., 2004  Strohmeier et al., 2006 |
| *Methanocaldococcus jannaschii* | 2YWJ | | 1.90 |  |
| * Pdx1-Pdx2 complex | |  | |  |
| ^ꝉ^ PfPdx2 in complex with PbPdx1 | |  | |  |

**Supplementary Figure S1.** Pyridoxal phosphate synthesis by PLP synthase. Pdx2 (bottom) converts L-glutamine into L-glutamate and ammonia. The ammonia molecule diffuses to the Pdx1 active site where it is converted together with D-ribose 5-phosphate and D-glyceraldehyde 3-phosphate into pyridoxal phosphate, a vitamer of vitamin B6.

**
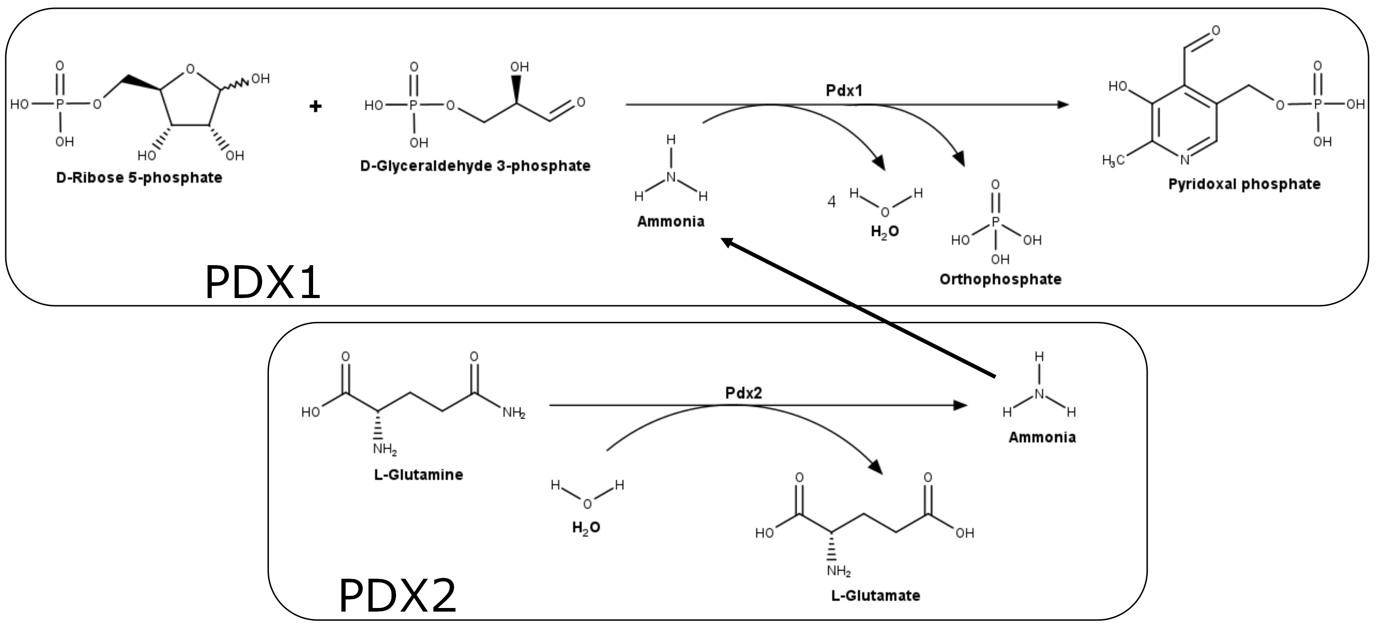
**

**Supplementary Video.** Pdx1 structural rearrangements during the catalytic cycle. The A. thaliana crystal structures shown in Figure 1G-1H were used in the Morph utility available in UCSF Chimera (Pettersen et al., 2004) to generate a video animation.

# References

Pettersen, E. F., Goddard, T. D., Huang, C. C., Couch, G. S., Greenblatt, D. M., Meng, E. C., et al. (2004). UCSF Chimera - A visualization system for exploratory research and analysis. *J. Comput. Chem.* 25, 1605–1612. doi:10.1002/jcc.20084.
